# Microglia-Specific Expression of *HEXA* and *HEXB* Leads to Poor Prognosis in Glioblastoma Patients

**DOI:** 10.3389/fonc.2021.685893

**Published:** 2021-08-04

**Authors:** Mengxian Jia, Wenbin Zhang, Junle Zhu, Changgang Huang, Jian Zhou, Jiashu Lian, Ying Wang, Honglin Teng, Zhihui Huang

**Affiliations:** ^1^College of Pharmacy, School of Medicine, Hangzhou Normal University, Hangzhou, China; ^2^Department of Orthopedics (Spine Surgery), The First Affiliated Hospital of Wenzhou Medical University, Wenzhou, China; ^3^Department of Neurosurgery, Tongji Hospital, Tongji University School of Medicine, Shanghai, China; ^4^Phase I Clinical Research Center, Zhejiang Provincial People’s Hospital of Hangzhou Medical College, Hangzhou, China

**Keywords:** glioblastoma, microglia, HEXA, HEXB, prognosis

## Abstract

Glioblastoma multiforme (GBM) is one of the deadliest cancers in brain. There have been few treatment advances for GBM despite increasing scientific understanding of this disease. β-hexosaminidase (Hex) is an important enzyme system in human body, encoded by two genes, *HEXA* and *HEXB*, are closely related to central nervous system (CNS) diseases such as Sandhoff disease (SD) and Tay-Sachs disease (TSD). However, the expression pattern and function of HEXA and HEXB in GBM remains unclear. Here, we found that both the mRNA and protein expression levels of HEXA and HEXB were significantly upregulated in GBM patient samples. The results from single-cell RNA-sequencing (scRNA-seq) database and double immunostaining showed that HEXA and HEXB were specifically expressed in microglia in GBM patient samples. Furthermore, our *in vitro* experiments revealed that conditioned media from *HEXA* and *HEXB* knockdown-microglia cells could inhibit the proliferation and migration of GBM cells. Finally, according to survival analysis based on online database, higher expression of *HEXA* and *HEXB* was associated with poor prognosis in GBM patients. In conclusion, these results suggest that microglial *HEXA* and *HEXB* play fundamental role in GBM progression, and they will be potential biomarkers for GBM.

## Introduction

Glioblastoma, also known as glioblastoma multiforme (GBM), is the most prevalent malignant tumor of human brain (1). Although the annual incidence of GBM is 3.19 per 100, 000 individuals, the highly invasive and heterogeneous features of GBM as grade IV glioma leads to its poor prognosis (2, 3). The available treatment options for this tumor are currently limited. The standard treatment for GBM patients is surgical resection, followed by radiotherapy and administration of temozolomide (4). However, these treatments do not lead to desirable outcomes. Thus, exploring new molecular targets for inhibiting its incidence and progression is urged. Notably, with the development of sequencing technologies and bioinformatics analysis, an increasing number of biomarkers have been found for the diagnosis of GBM, which can lead to its timely detection and treatment (5). In humans, β-hexosaminidase (Hex) is an important lysosomal enzyme that degrades various cellular substrates, such as oligosaccharides, glycosaminoglycans and glycolipids (6). Under the physiological conditions, α- and β- subunits of Hex are encoded by two genes, *HEXA* and *HEXB*, respectively and these subunits dimerize to form β-hexosaminidase A (HexA; αβ) and β-hexosaminidase B (HexB; ββ) (7). Hex mediates the breakdown of GM2 ganglioside, and the loss of its function results in GM2 accumulation and progressive neurodegenerative diseases, such as Sandhoff disease (SD) and Tay-Sachs disease (TSD) (8, 9). In addition, studies have shown that Hex would accumulate and cause immune response in Alzheimer’s disease (AD) and Down syndrome (10). To sum up, mutations in *HEXA* and *HEXB* are closely related to diseases affecting central nervous system (CNS).

Evidence supporting the role of *HEXA* and *HEXB* in tumor progression has been accumulating. In laryngeal cancer, Hex has recently been identified as a new potential biomarker. In this cancer, Hex might be involved in the release of a particular sugar from the ends of oligosaccharide chains of glycocalyx proteins, which changes the adhesive forces that bind the cells together as well as the communication between the cells and extracellular matrix and leads to enhance migration capacity of tumor cells (11). Another study has reported that Hex might be an useful general marker for detecting the tumor burden or a more specific marker for liver metastases (12). Using the single-cell RNA-sequencing (scRNA-seq) technique, it has been reported that Hex is likely expressed in the microglia (13). Further, it has been shown that microglia might promote the development of GBM by secreting various cytokines (14). However, the role of *HEXA* and *HEXB* in the onset and progression of GBM as well as effects of their expression in the microglia remain unclear.

In this study, using bioinformatics analysis and *in vitro* assays, we aimed to examine the role of HEXA and HEXB in progression of GBM and whether *HEXA* and *HEXB* could serve as potential prognostic biomarkers for GBM, which would lead to the identification of new targets for the treatment of GBM.

## Materials and Methods

### Oncomine Database Analysis

Oncomine database (http://www.oncomine.org/), a cancer microarray database and web-based data-mining platform for new discovery from genome-wide expression analyses, was used to analyze the *HEXA* and *HEXB* mRNA expression between GBM and normal tissues in humans. For this study, the thresholds were set as follows: fold change was defined as 2 and p-value was set at 0.05.

### Chinese Glioma Genome Atlas (CGGA) Database Analysis

CGGA (http://www.cgga.org.cn) is an online database for analyzing brain tumor datasets from Chinese cohorts. This database was used to browse *HEXA* and *HEXB* mRNA expression profiles and perform survival analysis for each glioma subtype. The hazard ratios with 95% confidence interval and log-rank p-values were computed.

### Gene Expression Profiling Interactive Analysis (GEPIA2)

GEPIA2 database (http://gepia2.cancer-pku.cn/) is an online database for analyzing several kinds of tumor samples from Cancer Genome Atlas (TCGA) and Genotype-Tissue Expression (GTEx) projects. This database was also used to perform survival analysis of glioma patients. The hazard ratios with 95% confidence interval and log-rank p-values were also computed.

### Single Cell Portal Database Analysis

The single cell portal (https://singlecell.broadinstitute.org/) was developed to facilitate the sharing of scientific results and disseminate data generated from single-cell technologies. This database was used to explore the expression of *HEXA* and *HEXB* genes in different types of brain cells.

### GBM Patient Samples and Hematoxylin and Eosin (HE) Staining

The study was approved by the ethics committee of Tongji Hospital of Tongji University, China. Tissue samples were collected from GBM patients after obtaining their informed consent. After fixed in 4% paraformaldehyde (PFA), the normal brain and GBM tissues were dehydrated and embedded in paraffin. Tissue sections (2 μm thick) were deparaffinized, rehydrated, and stained with hematoxylin and eosin to observe the normal and tumor structures.

### Immunohistochemistry

Paraffin sections were dewaxed thrice in xylene (10 min each), and were then rehydrated with 100%, 95%, and 75% alcohol, rinsed in de-ionized water, and placed twice in 1X sodium citrate antigen retrieval solution in a microwave oven for antigen retrieval (10 min each). The sections were then washed with phosphate-buffered saline (PBS) and permeabilized with 0.3% Triton X-100 for 30 min, and blocked in 5% bovine serum albumin (BSA) for 30 min at room temperature. Sections were then incubated with primary antibody, including anti-HEXA (1:50, 11317-1-AP, Proteintech) and anti-HEXB (1:50, 16229-1-AP, Proteintech) antibodies at 4°C overnight. The following day, the sections were incubated with secondary antibodies for 60 min at room temperature. After washing with PBS, diaminobenzidine solution was added for signaling detection and brown color indicated positive signals. Finally, hematoxylin was used to counterstain, and the sections were then dehydrated using 75%, 95%, and 100% alcohol and xylene and sealed with neutral balsam. The sections were observed under Olympus SLIDEVIEW™ VS200 microscope (Olympus, Germany) and processed by Image J software.

### Immunofluorescence

The normal brain and GBM tissues were placed in 4% PFA for 2 days, and then dehydrated in 30% sucrose until they sank to the bottom, and then cut into 10 μm coronal sections using a Thermo freezing microtome. After placing in 1× sodium citrate antigen retrieval solution in a thermostatic bath at 98°C for 30 min, the sections were permeabilized and blocked with 0.3% Triton X-100 in PBS containing 5% BSA at room temperature for 1 h. Subsequently, sections were incubated in primary antibody, including anti-HEXA (1:50, 11317-1-AP, Proteintech), HEXB (1:50, 16229-1-AP, Proteintech) and TMEM119 (1:100, 66948-1-Ig, Proteintech) antibodies at 4°C overnight. Sections were washed three times with PBS and then with incubated in 1:1000 diluted solution of secondary antibody (A11035/A11001, Invitrogen) at room temperature for 1 h. Finally, the sections were sealed with mounting medium. Images were acquired using a confocal microscope (FV3000; Olympus). The total density of fluorescence was measured using ImageJ software.

### cDNA Constructs

The HEXA shRNA (5′-CCGGCCCAGTCTCAACAGCACCTATCTCAGAGAATAGGTGCTGTTGAGACTGGGTTTTTTG-3′) and HEXB shRNA (5′-CCGGGGAAATTATTTCATCCTTAAACTCAAGAGATTTAAGGATGAAATAATTTCCTTTTTTG-3′) sequences were inserted into the pLKD-CMV-eGFP-U6-shRNA vector (OBiO Technology). The expression constructs were verified by sequencing.

### Plasmid Transfection and Cell Culture

The BV2 microglial cell line and human GBM cell line DBTRG were kindly gifted by Prof. Gang Chen’s laboratory (Zhejiang University, China) and were grown in Dulbecco’ s modified Eagle’s medium (DMEM, Gibco) supplemented with 10% fetal bovine serum (FBS, Gibco) and 1% penicillin/streptomycin (Gibco), at 37°C in a 5% CO_2_ atmosphere. When BV2 cell density in the six-well plates reached 80%, cells were transfected shRNA constructs by using Lipofectamine 3000 transfection reagent (L3000-015, Invitrogen) for 24 h. Subsequently, the incubation medium was removed, and serum-free DMEM was added for another 24 h. Finally, the conditioned media was collected and centrifuged to remove any floating cells, and was then used in subsequent experiments.

### Western Blotting

Briefly, cultured cells were lysed by cold RIPA Buffer and incubated at 4°C for 30 min, followed by 20 min centrifuge at 12, 000 ×g. The protein concentrations were quantified with a BCA Protein Assay kit (Thermo Fisher Scientific), then proteins were extracted with 5× loading buffer and boiled at 100°C water for 10 min, and were then separated by using 10% sodium dodecyl sulphate-polyacrylamide gel electrophoresis (SDS-PAGE) and were transferred onto a polyvinylidene fluoride (PVDF) membrane (Merck Millipore). Following blocking with 5% non-fat dry milk for 1 h, the membranes were incubated with HEXA (1:1, 000, 11317-1-AP, Proteintech), HEXB (1:1, 000, 16229-1-AP, Proteintech) and GAPDH (1:10, 000, 200306-7E4, ZEN BIO) antibodies overnight at 4°C. After washing for 3 times in TBST, the membranes were then incubated with secondary antibodies at room temperature for 1 h. After washing in TBST for another three times, the protein signals were detected using electro-chemiluminescence imaging analysis system (GelViev 6000Plus, BLT). Blots were measured by Image J software.

### Wound Healing Assay

For the wound healing assay, DBTRG cells were cultured in six-well plates until the cell confluence reached 90%. A wound was created in each plate with a 200 µL plastic pipette tip. Then, the collected conditioned medium from HEXA and HEXB knockdown-BV2 cells or control cells was added to six-well plate. Images were acquired using light microscopy (CKX53; Olympus) at 0 h and 24 h after scratching, and the migration rate was calculated by measuring the width of the wound.

### Colony Formation Assay

DBTRG cell suspension (2, 000 cells/plate) was seeded onto six-well plates containing 3 mL conditioned medium from HEXA and HEXB knockdown-BV2 cells or control cells. After 1-week, visible clones were observed in the culture plates. Subsequently, colonies were fixed and stained with gentian violet for 30 min and washed 2 times with PBS.

### Statistical Analysis

GraphPad Prism 8 was used for statistical analysis. Student’s t test or one-way analysis of variance (ANOVA) was used for comparing two independent groups. p<0.05 was considered statistically significant.

## Results

### *HEXA* and *HEXB* mRNA Levels Are Upregulated in GBM Patient Samples

**Figure 1:**
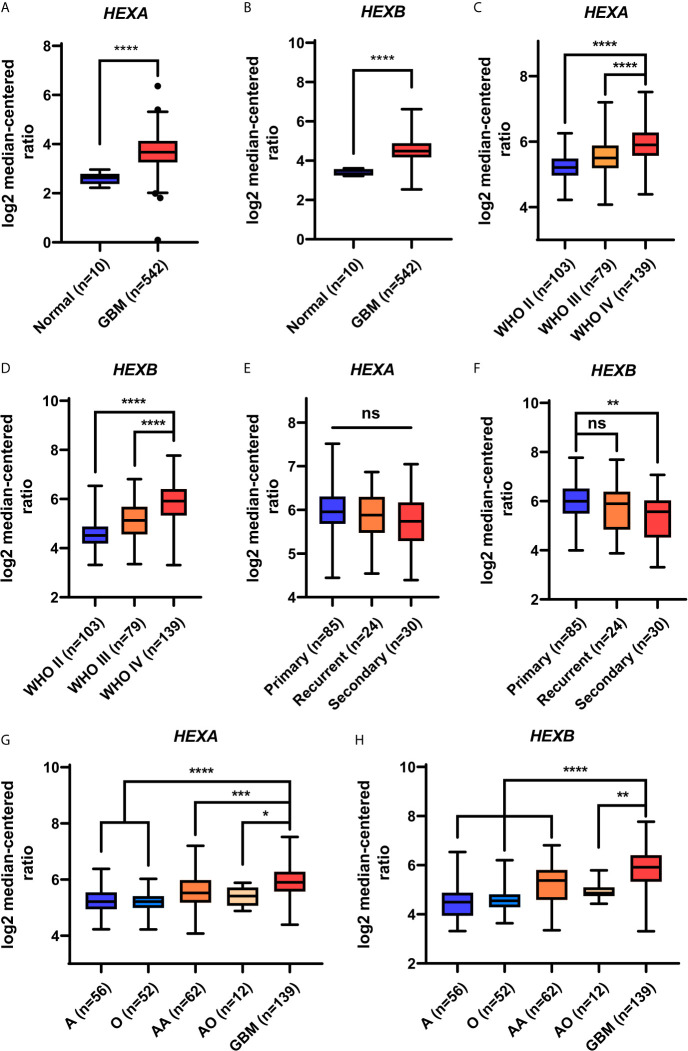
Upregulation of mRNA levels of *HEXA* and *HEXB* in GBM based on Oncomine and CGGA database analysis. **(A, B)** Analysis of *HEXA* and *HEXB* mRNA expression in the Oncomine database (t-test, GBM *vs* Normal). **(C–H)** Analysis of *HEXA* and *HEXB* mRNA expression in the CGGA database (one-way ANOVA, WHO IV *vs* WHO II and WHO III, Primary *vs* Recurrent and Secondary, GBM *vs* A, O, AA and AO). A (diffuse astrocytoma), O (oligoastrocytoma), AA (anaplastic astrocytoma), AO (anaplastic oligoastrocytoma), GBM (glioblastoma). ^*^ *p* < 0.05, ^**^ *p* < 0.01, ^***^ *p* < 0.001, ^****^ *p* < 0.0001, ns, not significant.

To explore the potential roles of HEXA and HEXB in GBM, the expression level of HEXA and HEXB in normal brain and GBM tissues was examined based on Oncomine database and the Cancer Genome Atlas (TCGA) database. As shown in **Figures 1A, B**, *HEXA* and *HEXB* mRNA levels were significantly upregulated in GBM tissues, compared with that in normal brain tissues. Interestingly, CGGA database showed that the expression level of HEXA and HEXB was higher in the highest-grade glioma (WHO IV), than that in low-grade gliomas (**Figures 1C, D, G, H**). However, no apparent statistical differences in mRNA levels of *HEXA* and *HEXB* between the primary, recurrent, and secondary groups were observed (**Figures 1E, F**). These results suggest that mRNA levels of *HEXA* and *HEXB* are upregulated in GBM and that their expression level is positively correlated with the grade of glioma.

### HEXA and HEXB Protein Levels Are Upregulated in GBM

**Figure 2:**
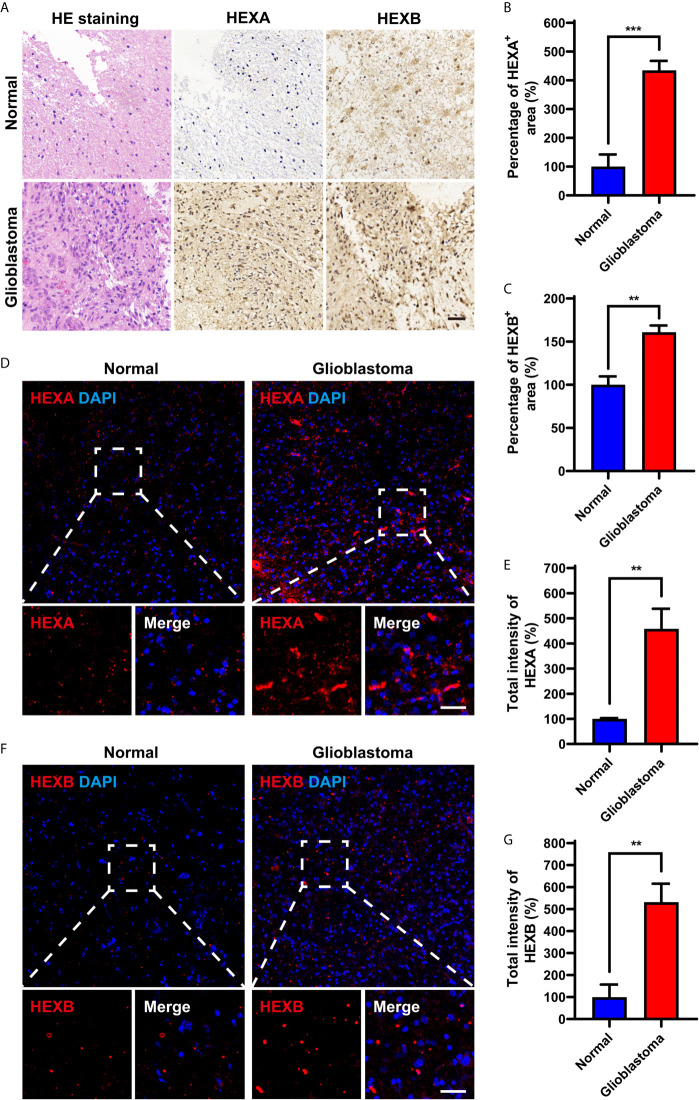
Upregulation of HEXA and HEXB protein levels in GBM patient samples. **(A)** HE staining and immunohistochemistry staining of HEXA and HEXB in normal tissues and GBM tissues. **(B, C)** Quantification of the percentage of HEXA^+^ and HEXB^+^ areas in images presented in A (n = 3) (t-test, GBM *vs* Normal). **(D)** Immunofluorescence staining of HEXA in normal tissues and GBM tissues. **(E)** Quantification of the intensity of HEXA as shown in **(D)** (n = 3) (t-test, GBM *vs* Normal). **(F)** Immunofluorescence staining of HEXB in normal tissues and GBM tissues. **(G)** Quantification of the total intensity of HEXB as shown in F (n = 3) (t-test, GBM *vs* Normal). HE (hematoxylin and eosin), GBM (glioblastoma). Scale bars = 50 μm. Data are presented as the mean ± SD. ^**^ *p* < 0.01, ^***^ *p* < 0.001.

We further examined the expression patterns of HEXA and HEXB proteins in the tissue samples obtained from GBM patients. As shown in **Figures 2A–C**, immunohistochemistry revealed that the percentage of HEXA- and HEXB-positive staining areas in GBM tissues was significantly higher than that in normal brain tissues. Furthermore, immunofluorescence staining further confirmed these results, and the total fluorescence intensity of HEXA and HEXB in GBM tissues was stronger than that in normal brain tissues (**Figures 2D–G**). Taken together, these results suggest that HEXA and HEXB protein levels are upregulated in GBM patient samples.

### HEXA and HEXB Are Upregulated in Microglia of GBM Patient Samples

**Figure 3:**
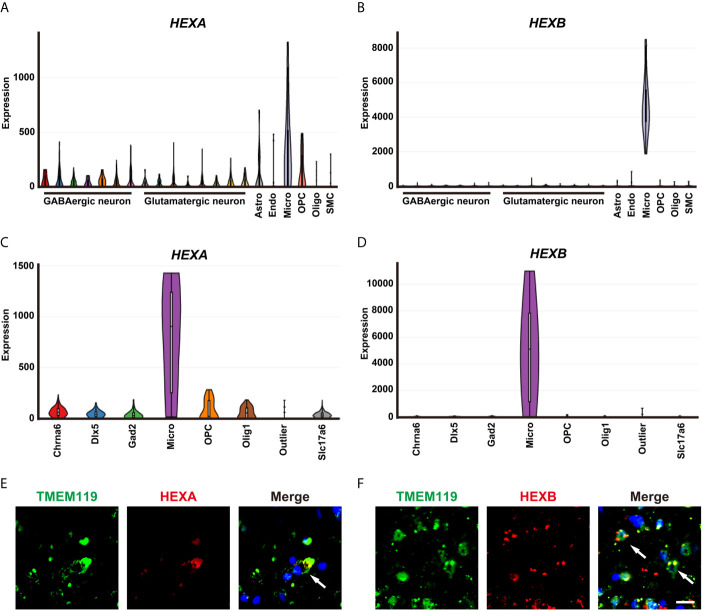
Upregulation of HEXA and HEXB in microglia of GBM patient samples. **(A, B)** Tasic’s scRNA-seq showed that mRNA levels of *HEXA* and *HEXB* in microglia were higher than those in other cell types in the nervous system. **(C, D)** The Allen Institute for Brain Science RNA-seq transcriptomic profiling showed that mRNA levels of *HEXA* and *HEXB* in microglia were higher than those in other cell types in the nervous system. **(E, F)** Double immunostaining of HEXA/HEXB (red) and TMEM119 (green) in GBM patient samples. Astro (Astrocytes), Endo (Endothelial cells), Micro (Microglia), OPC (Oligodendrocyte precursor cells), Oligo (Oligodendrocyte), SMC (Smooth muscle cells), Chrna6 (Cholinergic neuron), Dlx5 (Neural stem cell), Gad2 (GABAergic neurons), Slc17a6(Glutamatergic neurons). Scale bars = 20 μm.

A single-cell portal database was used to explore the cellular distribution of HEXA and HEXB in GBM patient samples. Based on the previous studies (13) and Allen Institute for brain science RNA-seq transcriptomic profiling (https://portal.brain-map.org/), we found that *HEXA* and *HEXB* mRNAs were mainly expressed in microglia. mRNA levels of HEXA and HEXB in microglia were higher than those observed for other cell types in the nervous system (**Figures 3A–D**). Indeed, further double immunostaining showed that HEXA and HEXB were mainly expressed in TMEM119^+^ microglia cells (**Figures 3E, F**). Taken together, these results suggest that HEXA and HEXB are upregulated in microglia of GBM patient samples.

### Conditioned Media From HEXA and HEXB shRNA-Microglia Treatment Inhibit the Proliferation and Migration of DBTRG Cells Through Secreting Some Factors

**Figure 4:**
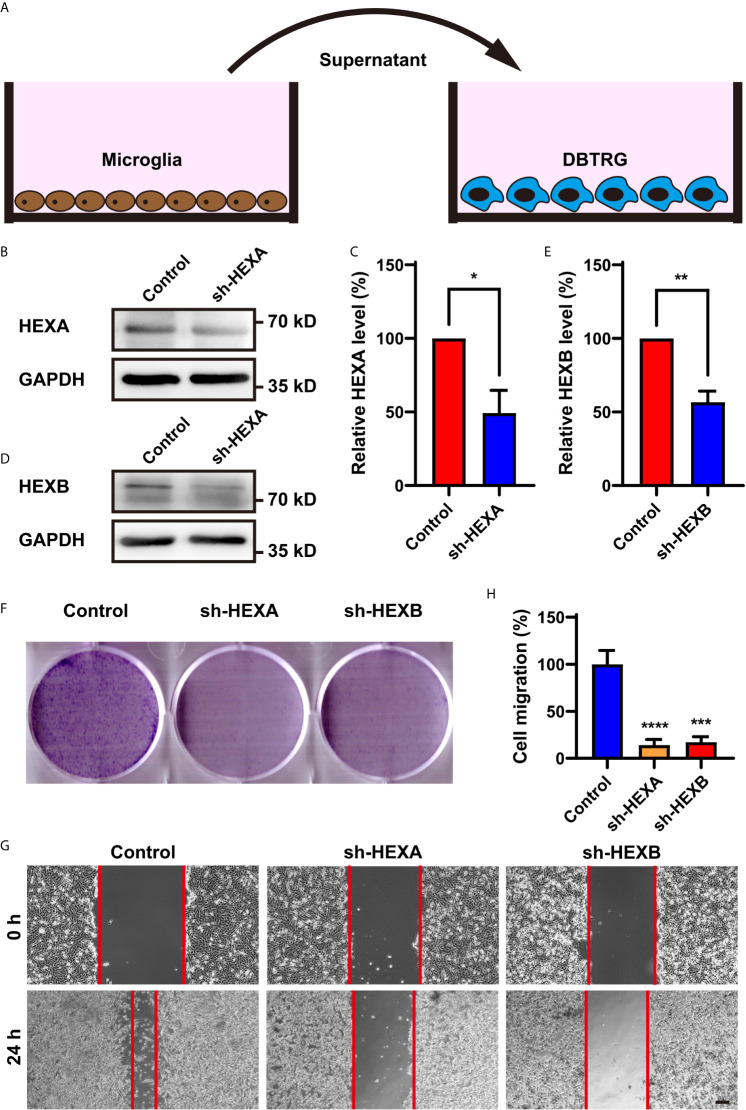
Conditioned media from HEXA and HEXB-knockdown-microglia cells inhibited the proliferation and migration of DBTRG cells. **(A)** Schematic diagram of the *in vitro* experiments. **(B)** Western blot analysis of knockdown efficiency of HEXA. **(C)** Quantification of HEXA expression as shown in **(B)** (n = 3) (t-test, sh-HEXA *vs* Control). **(D)** Western blot analysis of knockdown efficiency of HEXB. **(E)** Quantification of HEXB expression as shown in **(B)** (n = 3) (t-test, sh-HEXB *vs* Control). **(F)** Representative images of DBTRG cells treated with conditioned media from control, HEXA and HEXB-sh-microglia cells in the colony formation assay. **(G)** Representative images of DBTRG cells treated with conditioned media from control, HEXA and HEXB-sh-microglia cells in the wound healing assay. **(H)** Quantified analysis of migration of DBTRG cells (n = 3) (one-way ANOVA, Control *vs* sh-HEXA and sh-HEXB). Scale bars = 100 μm. Data are presented as the mean ± SD. ^*^ *p* < 0.05, ^**^ *p* < 0.01, ^***^ *p* < 0.001, ^****^ *p* < 0.0001.

To explore the function of microglial HEXA and HEXB in GBM, we performed *in vitro* experiments. The shRNA of HEXA and HEXB were constructed and their RNAi efficiency was confirmed by western blotting in BV2 microglia cells (**Figures 4B–E**). To further examine whether microglial HEXA and HEXB have function on tumorigenesis of GBM in a paracrine manner, conditioned media from HEXA and HEXB shRNA-microglia cell lines was collected and then treat to DBTRG cells, a cell line of GBM (**Figure 4A**). As shown in **Figures 4F–H**, conditioned media from HEXA and HEXB shRNA-microglia treatment significantly reduced the migration of DBTRG cells in the wound healing assay, and also inhibited the proliferation of DBTRG cells in the colony formation assay, compared with control conditioned media. These results suggest that microglial HEXA and HEXB promote the migration and proliferation of DBTRG cells through secreting some factors.

### High *HEXA* and *HEXB* mRNA Expression Is Associated With Poor Prognosis in GBM Patients

**Figure 5:**
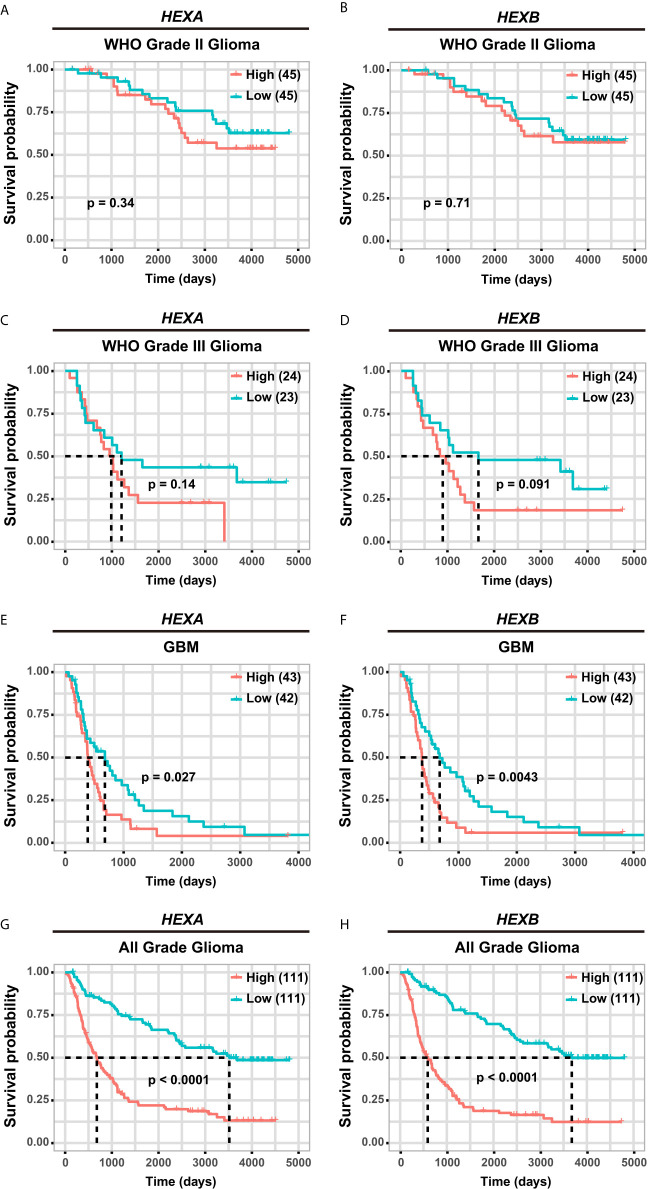
Higher expression of *HEXA* and *HEXB* was associated with poor prognosis in GBM patients according to CGGA database. **(A, B)** Survival analysis of WHO grade II glioma patients related with *HEXA* and *HEXB* expression. **(C, D)** Survival analysis of WHO grade III glioma patients related with *HEXA* and *HEXB* expression. **(E, F)** Survival analysis of GBM patients with *HEXA* and *HEXB* expression. **(G, H)** Survival analysis of glioma patients of all grades *related with HEXA* and *HEXB* expression. (survival analyses, High *vs* Low), *p*<0.05 was considered statistically significant.

**Figure 6:**
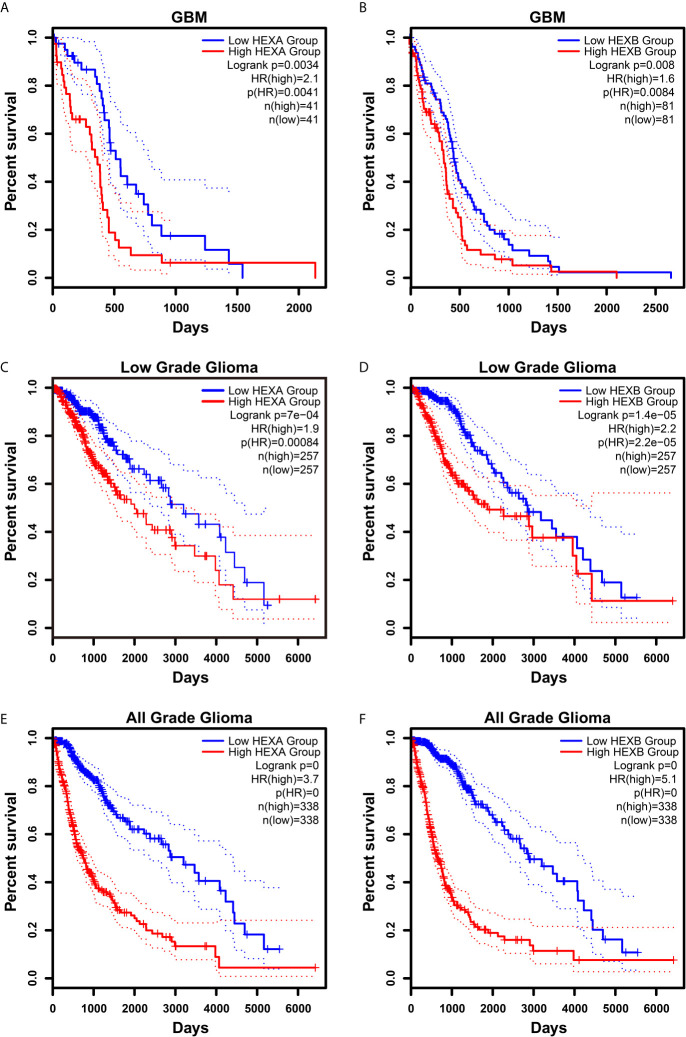
Higher expression of *HEXA* and *HEXB* was associated with poor prognosis in GBM patients according to GEPIA2 database. **(A, B)** Survival analysis of GBM gliomas patients related with *HEXA* and *HEXB* expression. **(C, D)** Survival analysis of low grade glioma patients related with *HEXA* and *HEXB* expression. **(E, F)** Survival analysis of glioma patients of all grades related with *HEXA* and *HEXB* expression. (survival analyses, High *vs* Low), *p*<0.05 was considered statistically significant.

Although according to the survival analysis based on CGGA database, no correlation was observed between *HEXA* and *HEXB* mRNA expression and the survival probability in grade II and III gliomas (**Figures 5A–D**), the survival probability of GBM patients with higher expression of *HEXA* and *HEXB* mRNA was significantly shorter (**Figures 5E, F**), suggesting that *HEXA* and *HEXB* expression might act as a prognostic marker in grade IV patients. Interestingly, all WHO grades of gliomas patients with higher *HEXA* and *HEXB* expression were predicted a poor prognosis (**Figures 5G, H**). These results were confirmed in GEPIA2 database. GBM patients with higher expression of *HEXA* and *HEXB* mRNA usually had shorter survival probability (**Figures 6A, B**). Meanwhile, other grade gliomas patients with higher expression of *HEXA* and *HEXB* mRNA expression were also predicted the lower survival probability (**Figures 6C–F**). Taken together, these results suggest that higher expression of *HEXA* and *HEXB* mRNA is associated with poor prognosis in GBM patients.

## Discussion

In the present study, we found that HEXA and HEXB were highly expressed in the microglia of GBM tissues and could promote the progression of GBM by secreting some factors. Furthermore, high expression of HEXA and HEXB was correlated with a poor prognosis in the patients with GBM. These findings indicate that the HEXA and HEXB could serve as a potential biomarker for GBM. This study explored the influence of microglia-specific expression of *HEXA* and *HEXB* in GBM for the first time.

With the development of RNA sequencing and bioinformatics analysis, many molecular biomarkers for tumors have been discovered in recent years. Although a huge database of GBM biomarkers belonging to various classes has been established lately, the validation of the efficiency of certain biomarkers remains challenging. GBM is a very aggressive and rapidly growing malignancy and is characterized by a poor prognosis. Therefore, the search for a new potential molecular biomarker, which can help in the development of potential remedies for this disease in terms of reversing the effects of cancer growth or suppressing the disease progression, is necessary. Sasmita et al. have identified the advantages and disadvantages of major biomarkers of GBM, such as O6-methylguanine-DNA methyltransferase, epidermal growth factor receptor, platelet-derived growth factor alpha receptor, and isocitrate dehydrogenase (5). Despite decades of research to develop an effective biomarker for the diagnosis and prognosis of GBM, only a few have shown promising results. Consistent with these previous studies, based on the literature and database search, in this study, we performed a bioinformatics analysis to assess whether HEXA and HEXB could serve as potential diagnostic and prognostic biomarkers for GBM. Oncomine database queries of the TCGA and CGGA datasets were analyzed to validate our speculation. Furthermore, immunohistochemistry and immunofluorescence staining also showed that HEXA and HEXB proteins were highly expressed in the GBM tissue. Simultaneously, survival analysis based on CGGA datasets suggested that higher *HEXA* and *HEXB* expression was associated with a poor prognosis in GBM patients. Therefore, these results suggests that HEXA and HEXB could be regarded as potential diagnostic and prognostic biomarkers for both primary and secondary GBM.

Further, using single-cell RNA sequencing and double immunostaining, we found that HEXA and HEXB were mainly expressed in microglial cells, which was consistent with a previous study has also reported HEXB to be a new marker of microglial cells in the brain (15). An increasing number of studies have acknowledged that GBM is a complex tumor composed of neoplastic and non-neoplastic cells and that each type of non-neoplastic cell, including fibroblasts, immune cells, and endothelial cells, may contribute to cancer formation and progression. These non-neoplastic cells can produce growth and survival factors, chemokines, extracellular matrix constituents, and angiogenic molecules to change the local milieu in which neoplastic cells grow and infiltrate (14). In fact, 30%–50% of the non-neoplastic cells in GBM are microglia or macrophages, and they are rich sources of these stromal factors (16, 17). Accumulating evidence indicates that microglia promote glioma growth and migration of GBM cells. One study has shown that the proportion of glioma cells increased by 3-fold in the presence of microglia *in vitro* (18). Microglia synthesize and release stress-inducible protein 1 (STI1), a cellular prion protein ligand that can increase the proliferation and migration of GBM cells *in vitro* as well as *in vivo* (19). In addition, microglial cells release growth factors, such as epidermal growth factor (EGF), which also stimulates GBM cell migration and invasion (20, 21). Another study has reported that transforming growth factor-β (TGF-β) also increases the migration of glioma cells through increase of integrin expression and function (22, 23). Consistent with these previous studies, our present study found that microglial HEXA and HEXB may regulates the secretion of some factors to promotes the proliferation and migration of GBM cells *in vitro*. Therefore, our results suggest that HEXA and HEXB might lead to the development of GBM by enhancing the proliferation and migration of the GBM cells by section of factors. In future, it needs to further study that what secreted factors are regulated by microglial HEXA and HEXB to promote tumorigenesis of GBM cells.

However, a few limitations can be picked from this study. Firstly, although we constructed shRNA of HEXA and HEXB and their RNAi efficiency was confirmed by western blotting in BV2 microglia cells, the efficiency of transfection needs further improvement by using other tool such as viruses. Secondly, in this study, we didn’t explore the detailed mechanism how HEX proteins affect the development of GBM. Thirdly, our studies are lack of *in vivo* functional experiments. In future, we will perform additional experiments to address these questions.

In conclusion, our results suggest that microglial HEXA and HEXB regulate the tumorigenesis of GBM through promoting their proliferation and migration, and may act as potential biomarkers for GBM.

## Data Availability Statement

The original contributions presented in the study are included in the article/supplementary material. Further inquiries can be directed to the corresponding authors.

## Ethics Statement

The studies involving human participants were reviewed and approved by Ethics Committee of Tongji Hospital of Tongji University, K-W-2021-002. The patients/participants provided their written informed consent to participate in this study.

## Author Contributions

MJ and WZ performed the research study and wrote this manuscript. JLZ, CH, JZ and JL supported during performing of the experiments, collected and analyzed data. YW, HT and ZH designed the research and provided experimental resource. All authors contributed to the article and approved the submitted version.

## Funding

This work was supported by the National Natural Science Foundation (81771348, 82071387).

## Conflict of Interest

The authors declare that the research was conducted in the absence of any commercial or financial relationships that could be construed as a potential conflict of interest.

## Publisher’s Note

All claims expressed in this article are solely those of the authors and do not necessarily represent those of their affiliated organizations, or those of the publisher, the editors and the reviewers. Any product that may be evaluated in this article, or claim that may be made by its manufacturer, is not guaranteed or endorsed by the publisher.
